# A common metric magnitude system for the perception and production of numerosity, length, and duration

**DOI:** 10.3389/fpsyg.2013.00449

**Published:** 2013-07-22

**Authors:** Virginie Crollen, Stéphane Grade, Mauro Pesenti, Valérie Dormal

**Affiliations:** ^1^Institut de Recherche en Sciences Psychologiques, Université catholique de LouvainLouvain-la-Neuve, Belgium; ^2^Institute of Neuroscience, Université catholique de LouvainBruxelles, Belgium

**Keywords:** magnitude processing, numerosity, length, duration, estimation

## Abstract

Numerosity, length, and duration processing may share a common functional mechanism situated within the parietal cortex. A strong parallelism between the processing of these three magnitudes has been revealed by similar behavioral signatures (e.g., Weber–Fechner's law, the distance effect) and reciprocal interference effects. Here, we extend the behavioral evidence for a common magnitude processing mechanism by exploring whether the under- and overestimation patterns observed during numerical perception and production tasks are also present in length and duration perception and production. In a first experiment, participants had to perform two estimation tasks (i.e., perception and production) on three magnitudes (i.e., numerosities, lengths, and durations). The results demonstrate similar patterns for the three magnitudes: underestimation was observed in all perception tasks, whereas overestimation was found in all production tasks. A second experiment ensured that this pattern of under- and over-estimation was not solely generated by the mere process of perceiving or producing something. Participants were required to estimate the alphabetical position of a letter (i.e., perception task) or to produce the letter corresponding to a given position (i.e., production task). No under- or overestimation were observed in this experiment, which suggests that the process of perceiving or producing something alone cannot explain the systematic pattern of estimation observed on magnitudes. Together, these findings strengthen the idea that magnitude estimations share a common metric system, requiring similar mechanisms and/or representations.

## Introduction

Number, space and time are fundamental properties of the environment constantly used by humans and animals to adapt and regulate their behavior to the external world. The idea of a unique functional mechanism supporting magnitude processing was primary developed for numerosity and duration in the Accumulator model (Meck and Church, 1983) and later extended by A Theory Of Magnitude (ATOM; Walsh, 2003; Bueti and Walsh, 2009). This model proposes the existence of a generalized magnitude processing system that would underlie the representation of numerosity, space and time through a common metric system controlled by areas of the parietal cortices. Three series of arguments support this idea.

At the neurofunctional level, brain areas located along the right intraparietal sulcus (IPS) are involved in numerosity, length, and duration discrimination. The involvement of these areas has been highlighted in neuroimaging (e.g., Pinel et al., 2004; Cohen Kadosh et al., 2005; Bueti and Walsh, 2009; Dormal and Pesenti, 2009; Dormal et al., 2012b), TMS (e.g., Bjoertomt et al., 2002; Alexander et al., 2005; Dormal et al., 2012a; Hayashi et al., 2013) and monkey electrophysiological (e.g., Leon and Shadlen, 2003; Nieder and Miller, 2003; Roitman et al., 2007; Tudusciuc and Nieder, 2007) studies.

At the developmental level, several studies have shown that discriminating numerosities, surface areas and durations leads to similar patterns of performance in babies (see respectively, Xu and Spelke, 2000; Brannon et al., 2006; van Marle and Wynn, 2006; for a review, see Feigenson, 2007): 6-month old infants are able to discriminate the numerosity, the duration or the size of one or several visual or auditory presented elements with a 1:2 ratio, but they fail to discriminate them with a 2:3 ratio. By 9 or 10 months, the precision of the representations improves, and infants develop the ability to discriminate durations and numerosities with a 2:3 ratio (Lipton and Spelke, 2003; Brannon et al., 2007). These studies therefore suggest that the discrimination of magnitude improves with development in a similar way for different magnitudes.

Finally, at the behavioral level, various similarities have been reported between the discrimination of numerosities, lengths, and durations. First, discriminating all three magnitudes obey Weber–Fechner's law (Stevens and Greenbaum, 1966; Teghtsoonian and Teghtsoonian, 1978), according to which the increase in stimulus intensity required to produce a noticeable increase of sensation is a constant function of the intensity of this stimulus^1^ (Fechner, 1860). Second, the behavioral signatures of the distance and size effects typically encountered in numerical judgments also appear when comparing other magnitudes. The distance effect refers to the observation that the ability to discriminate two numbers increases as the numerical distance between them increases (Moyer and Landauer, 1967; Buckley and Gillman, 1974). The size effect reflects the fact that, at equal numerical distance, the discrimination of two numbers decreases as their numerical size increases (Restle, 1970; van Oeffelen and Vos, 1982). Both effects are present in most judgments of quantifiable physical dimensions such as line lengths (e.g., Henmon, 1906; Johnson, 1939; Fias et al., 2003; Dormal and Pesenti, 2007), duration of sequences (e.g., Droit-Volet et al., 2004), physical size of geometric forms (e.g., Fulbright et al., 2003), and physical size of numerical symbols (e.g., Pinel et al., 2004; Cohen Kadosh et al., 2005; Kaufmann et al., 2005; Tang et al., 2006). Third, professional musicians, known to outperform non-musicians in temporal discrimination tasks, showed evidence of improved abilities also in spatial and numerical discrimination (Agrillo and Piffer, 2012). Fourth, several studies have investigated the influence of concurrent cognitive or motor tasks on numerosity, length, and duration processing. For example, the transient distortions in both space and time occurring after saccadic eye movements (i.e., compression of perceived magnitude of spatial separations and temporal intervals to approximately half of their true value; Morrone et al., 2005; Burr and Morrone, 2006) have recently also been reported during a numerosity perception task (Burr et al., 2010; Binda et al., 2011). The bisection of time, number and length were affected in a similar way by a click-train procedure (i.e., the presentation of a train of auditory clicks during a bisection judgment; Droit-Volet, 2010). Behavioral interactions between various quantifiable dimensions during estimation processing were also reported in interference paradigms exploring the influence of an irrelevant magnitude on the judgment of another magnitude (for a review, see Dormal and Pesenti, 2012). Whereas bidirectional interference effects were consistently observed between numerosity and space (e.g., Dormal and Pesenti, 2007; de Hevia and Spelke, 2009), the numerical cues influenced duration perception and reproduction but not the reverse (e.g., Dormal et al., 2006; Xuan et al., 2007; Agrillo et al., 2010; Chang et al., 2011; Vicario, 2011; but see Javadi and Aichelburg, 2012). Finally, a mutual interference was reported between time and space (e.g., Casasanto and Boroditsky, 2008). All these results suggest the existence of a continuum of automaticity, in which numerosity processing takes place more or less automatically, followed by length processing and then duration processing (Dormal and Pesenti, 2013).

Together, these behavioral similarity and interference results, and the common activation areas support Walsh's (2003) proposal of a generalized magnitude processing underlying the representation of numerosity, space, and duration. However, asymmetric interference results between numerical and temporal dimensions (Droit-Volet et al., 2004; Dormal et al., 2006; Agrillo et al., 2010) and the absence of numerical learning transfer to the discrimination of length (DeWind and Brannon, 2012) did not support the idea of a fully common magnitudes processing. Moreover, recent TMS and neuropsychological studies have revealed the presence of a double dissociation between numerosity and duration processing (Dormal et al., 2008, 2012c; Cappelletti et al., 2009, 2011). Two brain-damaged patients showed specific deficits in either numerical or duration processing (Cappelletti et al., 2009, 2011), while left parietal stimulation disrupted only numerosity processing in healthy participants (whereas duration processing was not impaired; Dormal et al., 2008). Finally, impairments in duration processing were observed both in elderly healthy adults and in patients suffering from early Parkinson's disease, whereas both groups performed correctly in a numerosity comparison task (Dormal et al., 2012c). These results suggest the coexistence of common and partially independent, rather than fully shared magnitude mechanisms and/or representations (Cappelletti et al., 2011; Dormal and Pesenti, 2012).

To explore the characteristics of the accuracy estimation profile observed during numerosity, length, and duration, perception and production tasks were used jointly for the first time in order to highlight similarities and differences in estimation processes. In perception tasks, non-symbolic stimuli, such as collections of dots, are presented to participants who have to estimate their numerosity by providing symbolic outputs, such as verbal or Arabic numerals. Conversely, in production tasks, participants produce non-symbolic numerosities (e.g., collection of dots or sequences of sounds) corresponding to symbolic stimuli (e.g., Arabic digits or verbal numerals). A specific pattern of behavioral results has been revealed in the literature on numerical cognition. While numerosities are systematically underestimated in perception tasks (Kaufman et al., 1949; Bevan and Turner, 1964; Krueger, 1972; Mandler and Shebo, 1982; Castronovo and Seron, 2007; Crollen et al., 2011), they are systematically overestimated in production tasks (Whalen et al., 1999; Cordes et al., 2001; Castronovo and Seron, 2007; Crollen et al., 2011; Crollen and Seron, 2012).

The present study was conducted to assess whether other magnitudes, such as length or duration, share or not a common metric system with numerosity, by determining whether under- and overestimation are also observed during length and duration perception and production tasks. In order to directly compare the performance patterns, Experiment 1 required each participant to perform both perception and production judgments on numerosity, length, and duration. If numerical, spatial and temporal estimations rely on a common mechanism and/or involve a common representation, the participants should underestimate these three magnitudes in the perception tasks, while a general overestimation should be observed in the production tasks. Dissimilarities in the performance patterns during the estimation of the three magnitudes would imply several core mechanisms using different metrics. To control whether those under- or overestimation patterns could be due to specific magnitude mechanisms and not to non-specific task requirement (perceiving or producing a stimulus), Experiment 2 was conducted with the same tasks but using unquantifiable material (i.e., letters).

## Experiment 1

### Method

#### Participants

Sixteen volunteers (5 males, 1 left-handed, mean age: 19.50 ± 0.97) took part in this experiment. They all had normal or corrected-to-normal vision and were unaware of the purpose of the study. All the procedures were non-invasive and were performed in accordance with the ethical standards laid down in the 1964 Helsinki Declaration.

#### Stimuli, tasks, and general procedure

**Figure 1:**
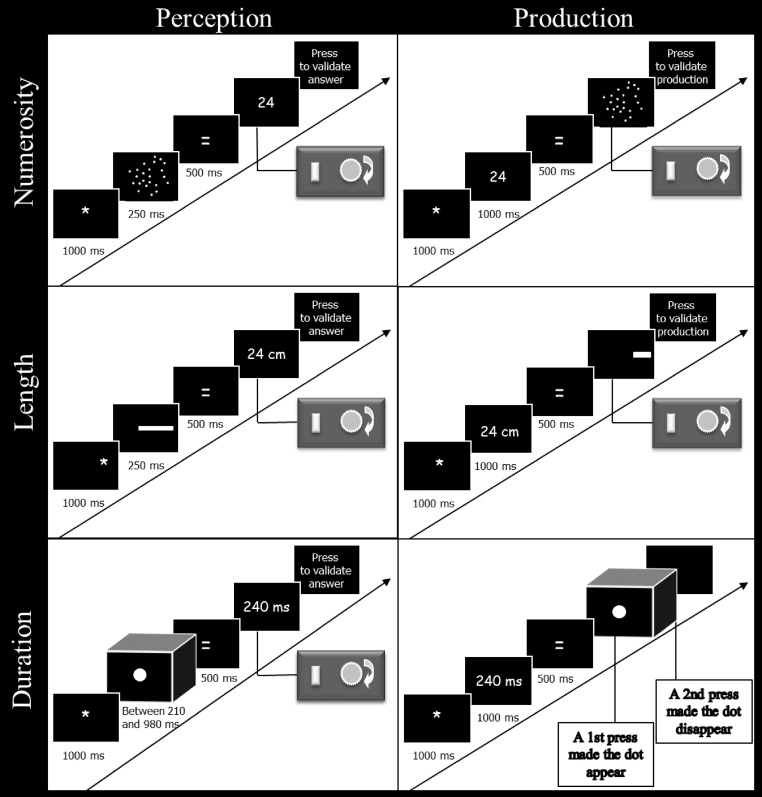
**Schematic representation of the time course of events used in the perception and production tasks for numerosity, length, and duration.** In the perception tasks, the participants had to estimate the numerosity, length, or duration of the stimuli by turning the potentiometer after the sign “=” had been displayed. In the production tasks, they had to produce a stimulus with a numerosity, length, or duration corresponding to the given value, by using the potentiometer after the sign “=” had been displayed. Responses were validated by pressing a button on the response box.

The participants had to perform two tasks (i.e., perception and production) on three magnitudes (i.e., numerosity, length, and duration), giving a total of six different conditions (Figure 1). The whole experiment lasted about 60 min and was divided into two sessions taking place on two different days. The three perception conditions were always performed during the first session, and the three production conditions during the second one. This procedure was adopted in order to prevent participants from being aware of the different values used for the perception tasks as the same values were explicitly presented in the production tasks. The order of magnitude processing within the first session was counterbalanced across participants and kept constant for the second session within participants.

The presentation procedure and the number of trials were identical in the six conditions. Each condition was composed of 3 blocks of 24 experimental trials, with every target values presented twice. Ten training trials were presented before the experimental trials in order to familiarize the participants with the tasks, and were not analyzed. The stimuli were projected on a screen measuring 1.65 m wide and 1.20 m high. This methodological choice was made to ensure that the participants could not use their knowledge of computer screen size to infer their answer and to allow a similar potential variability in the answers of the participants across the different dimensions. The participants sat 95 cm from the screen in a dark room. Stimulus presentation and data collection were controlled by a PC computer connected to a data projector and using a customized E-prime 2 program (Schneider et al., 2002).

The same range of values was used in the different conditions (21, 24, 28, 32, 37, 42, 49, 56, 64, 74, 85, 98 dots or cm for numerosity and length, respectively; 210, 240, 280, 320, 370, 420, 490, 560, 640, 740, 850, 980 ms for duration). These figures were chosen by applying a ratio between two consecutive values ranging from 0.85 to 0.93, as this ratio corresponds to the discrimination threshold in adults (Halberda and Feigenson, 2008). For numerosity, the range started at 21, as previous studies have shown that estimation is usually accurate for values below 20 (Indow and Ida, 1977; Krueger, 1982, 1984). Durations less than one second were presented to avoid explicit counting strategies. Lengths smaller than 1 m were chosen to reduce the use of perceptual cues such as the distance separating the target stimulus and the edge of the screen.

#### Perception conditions

***Numerosity.*** The participants had to estimate the numerosity of various visual arrays of white dots displayed on a black background. In order to control low-level continuous perceptive variables (Dehaene et al., 2005), two different arrays of dots were created: an extensive and an intensive sets. In the extensive set, the sum of the area of all the dots (i.e., the luminance) on the screen was kept constant across numerosities. As a consequence, the size of the dots decreased while the density of the array increased as numerosity increased. In the intensive set, the size of the dots and the density of the array were kept constant. Therefore, the luminance and the total occupied area increased with increasing numerosity.

Each trial began with the presentation of the sign “^*^” for 1000 ms. Then, an array of dots was flashed on the screen for a duration of 250 ms followed by the sign “=” (500 ms). After the sign “=,” the Arabic numeral “1” was presented, indicating to the participants that they had to give their estimation. To give their answers, participants had to turn a potentiometer on a response box (Mejias et al., 2012) to go through the Arabic numeral sequence. The potentiometer allowed the display of all the values between 1 and 255 (by jump of one unit every 1.4° of angular rotation). To validate their answers, the participants pressed a button on the response box.

***Length.*** The participants had to estimate the length (in cm) of horizontal rectangles. The rectangles were white, measured 4.5 cm in height and were presented on a black background. To avoid estimations based only on the distance from the edge of the screen, the rectangle occupied two different positions on the screen: on the left, where the rectangle began 10 cm from the left edge of the screen; or on the right, where the end of the rectangle was at 10 cm from the right edge of the screen. The sign “^*^” appeared either on the left or the right of the screen for 1000 ms for each trial, to warn the participant on which part of the screen the rectangle would be presented. Afterwards, a rectangle was displayed on the same side as the sign “^*^” for a duration of 250 ms, followed by the sign “=” centrally presented for 500 ms followed by the Arabic numeral “1.” This Arabic numeral indicated to the participants that they had to give their response by using the potentiometer (jump of one unit every 1.4° of angular rotation; the letters “cm” appeared next to the Arabic numerals). To validate their answers, the participants pressed a button on the response box.

***Duration.*** The participants had to estimate the duration of presentation of a dot. The trials started with the central presentation of the sign “^*^” (1000 ms). Then a white dot, 16 cm in diameter, was displayed on a black background in the center of the screen for a given duration (i.e., between 210 and 980 ms; see above for detailed values). After stimulus offset, the sign “=” appeared (500 ms), followed by the Arabic numeral “10” that indicated to the participants that they had to produce their answer by turning the potentiometer (the letters “ms” appeared next to the Arabic numerals). In this task, the use of the potentiometer induced a jump of 10 units every 1.4° of angular rotation and allowed the responses to range from 10 to 2550 ms. To validate their answers, the participants pressed a button on the response box.

#### Production conditions

***Numerosity.*** The participants had to produce an array of dots corresponding to the numerosity of a target Arabic numeral. Each trial started with the sign “^*^” presented for 1000 ms, followed by an Arabic numeral also presented for 1000 ms in the center of the screen. This Arabic numeral indicated the number of dots to produce. The sign “=” was then displayed on the screen for 500 ms followed by a single dot which indicated to the participants that they had to start their production by turning the potentiometer (with a jump of one dot for every 1.4° of angular rotation). The response box allowed the participants to produce all the numerosities ranging from 1 to 254. In a random way, half of the array of dots produced by the participants belonged to the intensive set while the other half belonged to the extensive set (see above for details). Finally, the participants pressed a button on the response box to validate their answers.

***Length.*** The participants had to produce a rectangle of a given length. Each trial started with the sign “^*^” presented for 1000 ms followed by an Arabic number for 1000 ms. The Arabic number indicated the length (in cm) of the rectangle to produce. Afterwards, the sign “=” appeared for 500 ms followed by a rectangle of 0.5 cm length which was displayed for half of the trials on the left of the screen (the start of the rectangle was 10 cm from the left edge of the screen; the rectangle was therefore generated from left to right), and, for the other half of the trials, on the right of the screen (the end of the rectangle was at 10 cm from the right edge of the screen; the rectangle was therefore generated from right to left). The participants had to turn the potentiometer to the right to increase or to the left to decrease the length of the rectangle (jump of 0.65 cm for every 1.4° of angular rotation). The maximum length of the rectangle that could be produced was 165 cm. To validate their answers, participants pressed a button on the response box.

***Duration.*** The participants had to produce time intervals. Each trial started with the sign “^*^” presented for 1000 ms, followed by an Arabic number for 1000 ms indicating the duration (ms) of the time interval to produce. Afterwards, the sign “=” was displayed on the screen for 500 ms, followed by an empty screen. In order to produce the time interval, participants had to press the button of the response box twice. The first press produced a central dot of 16 cm of diameter and indicated the beginning of the time interval; the second press made the dot disappearing and finished the production of the time interval. There was no time limit for the interval production.

#### Data analyses

For each target value and in every condition, any response which fell 2 or more standard deviations (*SD*) above the mean individual response was excluded from the analyses. As the mean estimates and their standard deviations were not normally distributed, logarithmic transformations were applied to the data before the following statistical analyses were carried out.

Firstly, to determine whether performance obeyed Weber–Fechner's law, linear mixed models (LMM) with magnitude (i.e., numerosity in the numerical estimation, length in the length estimation, and duration in the temporal estimation) as a fixed effect and participants as a random effect were conducted for perception and production conditions on log(mean) and log(*SD*).

Secondly, a mean error rate (ER) for each condition was calculated as follows: ER = [(participant's response—target value)/target value]^*^100. An ER of zero indicates accurate estimation, a negative ER indicates underestimation, and a positive ER indicates overestimation. The mean ER for each task and magnitude was then submitted to *t*-tests with a test value of 0 to determine the presence of significant under- or overestimation. In order to directly compare the three magnitudes, an ANOVA was carried out on the mean ER with condition (perception vs. production) and magnitude (numerosity, length vs. duration) as within-subject variables.

### Results

#### Linear mixed models on separate magnitudes

**Figure 2:**
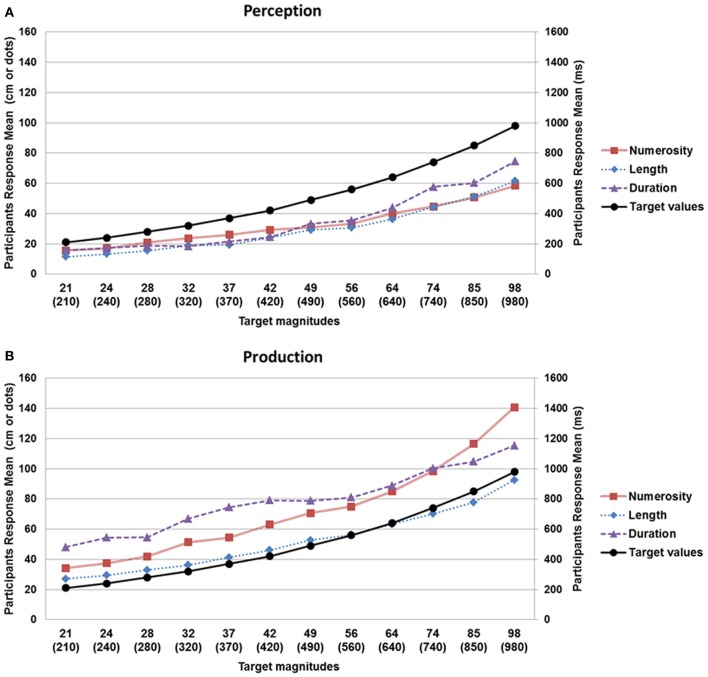
**Mean estimation values observed in the perception (A) and production (B) tasks as a function of target value (black line: target values; red line: numerosity; blue line: lenght; mauve line: duration).** The left vertical axis shows the estimated values for numerosity and length (in number of dots and cm, respectively) while the right axis corresponds to the values for duration (in ms). On the horizontal axis, the first row corresponds to numerosity (number of dots) or length (cm), while the values in brackets on the second line indicate duration (ms). As shown in the upper graph, a systematic underestimation was observed in the perception tasks, whereas an overestimation was present in the production tasks (lower graph), for the three magnitudes.

***Numerosity.*** For the perception condition, the results of the LMM showed that the log of the mean estimates increased with target numerosity, *F*_(11, 180)_ = 83.12, *p* < 0.001, as did the log of the standard deviation, *F*_(11, 180)_ = 17.80, *p* < 0.001, suggesting that the numerosity estimations obeyed Weber–Fechner's law; Figure 2A. For the production condition, the log of the mean estimates and the log of the standard deviation increased with target numerosity, *F*_(11, 180)_ = 70.13, *p* < 0.001 for log(mean); *F*_(11, 179)_ = 19.57, *p* < 0.001 for log(*SD*), Figure 2B.

***Length.*** For the perception condition, the log of the mean estimates and the log of the standard deviation increased with target value, *F*_(11, 180)_ = 69.15, *p* < 0.001 for log(mean); *F*_(11, 178)_ = 4.66, *p* < 0.001 for log(*SD*); Figure 2A. For the production condition, the log of the mean estimates and the log of the standard deviation were also found to increase with target value, *F*_(11, 180)_ = 121.39, *p* < 0.001 for log(mean); *F*_(11, 180)_ = 9.55, *p* < 0.001 for log(*SD*), suggesting that participants' production judgments obeyed Weber–Fechner's law; Figure 2B.

***Duration.*** For the perception condition, the results of the LMM showed that participants' judgments obeyed Weber–Fechner's law: the log of the mean estimates increased with duration, *F*_(11, 180)_ = 12.61, *p* < 0.001; the log of the standard deviation also increased with duration, *F*_(11, 180)_ = 8.25, *p* < 0.001; Figure 2A. For the production condition, the log of the mean estimates and the log of the standard deviation also increased with target value, *F*_(11, 180)_ = 9.23, *p* < 0.001 for log(mean); *F*_(11, 180)_ = 2.65, *p* < 0.01 for log(*SD*); Figure 2B.

#### Comparison across magnitudes

**Figure 3:**
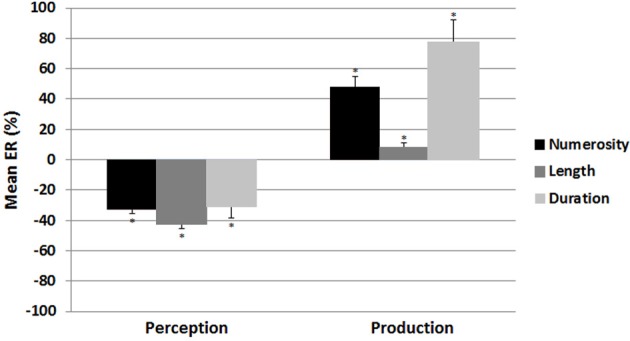
**Mean error rate as a function of condition (perception vs. production) and magnitude (numerosity, length, vs. duration) in Experiment 1.** A significant underestimation was observed in the perception tasks, whereas a significant overestimation was observed in the production tasks, for the three magnitudes. Error bars denote Standard Error of the Mean (SEM); asterisks indicate significant differences compared to 0.

In the perception conditions, a significant underestimation was observed for each magnitude as confirmed by the results of *t*-tests with the test value of 0; mean ER rates for numerosity: −32.4 ± 10.5%; *t*_(15)_ = −12.574, *p* < 0.001^2^ ; for length: −42.2 ± 12.9%; *t*_(15)_ = −13.037, *p* < 0.001; and for duration: −30.6 ± 31.8%; *t*_(15)_ = −3.846, *p* < 0.003; Figure 3. Similarly, in the production conditions, all the *t*-tests were significant, indicating the presence of overestimation in each magnitude; mean ER rates for numerosity: 47.82 ± 29.3%; *t*_(15)_ = 6.521, *p* < 0.001^3^ ; for length: 7.5 ± 14.5%; *t*_(15)_ = 2.081, *p* < 0.05; and for duration: 77.1 ± 61.7%; *t*_(15)_ = 4.996, *p* < 0.001; Figure 3.

The ANOVA performed on the mean ER with condition and magnitude as within subject-variables demonstrated a significant main effect of magnitude, *F*_(2, 30)_ = 23.9 *p* < 0.001: a significant difference was found between numerosity and length [*t*_(15)_ = 6.53, *p* < 0.01] and between length and duration [*t*_(15)_ = 6.31, *p* < 0.01], while the remaining comparison was not significant (*p* > 0.1). A main effect of condition, *F*_(1, 15)_ = 66.7 *p* < 0.001, indicated that the ER for the perception tasks (*M* = −35.3 ± 12.74) was lower than the ER for the production tasks (*M* = 44.1 ± 30.1). Since the interaction between condition and magnitude was also significant, *F*_(2, 30)_ = 6.675, *p* < 0.005, separate ANOVAs were conducted for perception and production conditions with magnitude as the within-subject variable. In the perception condition, no significant effect of magnitude was revealed, *F*_(2, 30)_ = 1.728, *ns*, suggesting that the underestimation rates were equivalent for the three magnitudes. In the production condition, a significant main effect of magnitude was observed, *F*_(2, 30)_ = 16.094, *p* < 0.001. The mean overestimation ER for length was smaller than that for numerosity or duration (all *p*-values < 0.001), that did not differ from each other (*p* > 0.1).

### Discussion of experiment 1

For all magnitudes and tasks, both the means and the standard deviations of the magnitude judgments increased with target value, and the variability in the participants responses was always proportional to the mean for a given target (i.e., the average magnitude of the error increased in proportion to the target), reflecting the scalar property (i.e., the signature of Weber–Fechner's law) already described in various magnitudes estimation tasks (e.g., Stevens, 1957; Meck and Church, 1983; Logie and Baddeley, 1987; Moyer and Landauer, 1967; Whalen et al., 1999).

Under- and over-estimation were observed during numerosity perception and production tasks, respectively, in line with the error pattern observed in previous studies on numerosity processing (e.g., Whalen et al., 1999; Cordes et al., 2001; Castronovo and Seron, 2007; Crollen et al., 2011; Crollen and Seron, 2012). These results were observed here in the particular condition of stimuli presentation on a large screen, suggesting that under- and over-estimation of numerosity occur whatever the conditions of presentation. Interestingly, this characteristic pattern is observed for the first time here in the estimation of length and duration. Participants' answers were indeed systematically underestimated in the three perception conditions, whatever the magnitude to process. Conversely, overestimation occurred whenever numerosity, length, or duration had to be produced. These findings clearly support the idea of a common metric system underlying the processing of numerosity, length, and duration (Walsh, 2003), and suggest that this common metric system could involve at least partially common mechanisms and/or representations. However, one cannot rule out the possibility that it is the processes of perceiving and/or producing something *per se* that cause the under- and overestimation, irrespective of the materials estimated. By using similar tasks (i.e., perception and production) but with an unquantifiable material (i.e., letters of the alphabet), Experiment 2 was specially designed to address this issue.

## Experiment 2

### Method

#### Participants

A total of 16 volunteers (8 males, mean age: 29 ± 7 years) participated after they gave their informed consent. They all had normal or corrected-to-normal vision, were unaware of the purpose of the study and had not participated in Experiment 1. All the procedures were non-invasive and were performed in accordance with the ethical standards laid down in the 1964 Helsinki Declaration.

#### Stimuli, tasks and procedure

As in Experiment 1, participants had to perform two tasks (i.e., perception and production), but with letters of the alphabet. The same letters were used in the two tasks; the first four and last four letters of the alphabet were not used as their positions could be overlearned, hence too easy to perceive/produce. Seven consonants and 4 vowels were chosen among the 18 remaining letters (i.e., E, F, H, I, K, N, O, R, T, U, V). Each task was composed of 1 block of 44 experimental trials with every letter presented four times. Stimulus presentation and data collection were controlled by a Dell laptop using a customized E-prime 2 program (Schneider et al., 2002) and equipped with a 15.6″ HD screen. The viewing distance was approximately 50 cm. The participant always began with the perception task; the whole session lasted about 15 minutes. The instructions of both perception and production tasks clearly mentioned to the participants that they had to estimate their answer and could not use counting strategies.

In the perception task, participants were required to give the alphabetical position of a letter (e.g., “*F*” is “6”). Each trial began with the presentation of the sign “^*^” for 1000 ms. Then, a letter was displayed on the screen for a duration of 1000 ms followed by the sign “=” (500 ms) indicating to the participants that they had to give their estimation. Participants were asked to answer aloud as fast and accurately as possible. We did not use the potentiometer in this task in order to avoid the use of a counting strategy that would probably have taken place if the letters had passed by one after the other. Response accuracy was monitored on-line by the experimenter. The next trial started immediately after the validation of the response by the experimenter (i.e., by pressing the space bar).

In the production condition, participants were asked to produce the letter corresponding to a given number (e.g., “6” is “*F*”). The presentation procedure was similar to the perception task, except that an Arabic number (corresponding to one of the positions of the 11 letters) was displayed on the screen.

#### Data analyses

The same data transformation and analyses as in Experiment 1 were applied.

### Results

**Figure 4:**
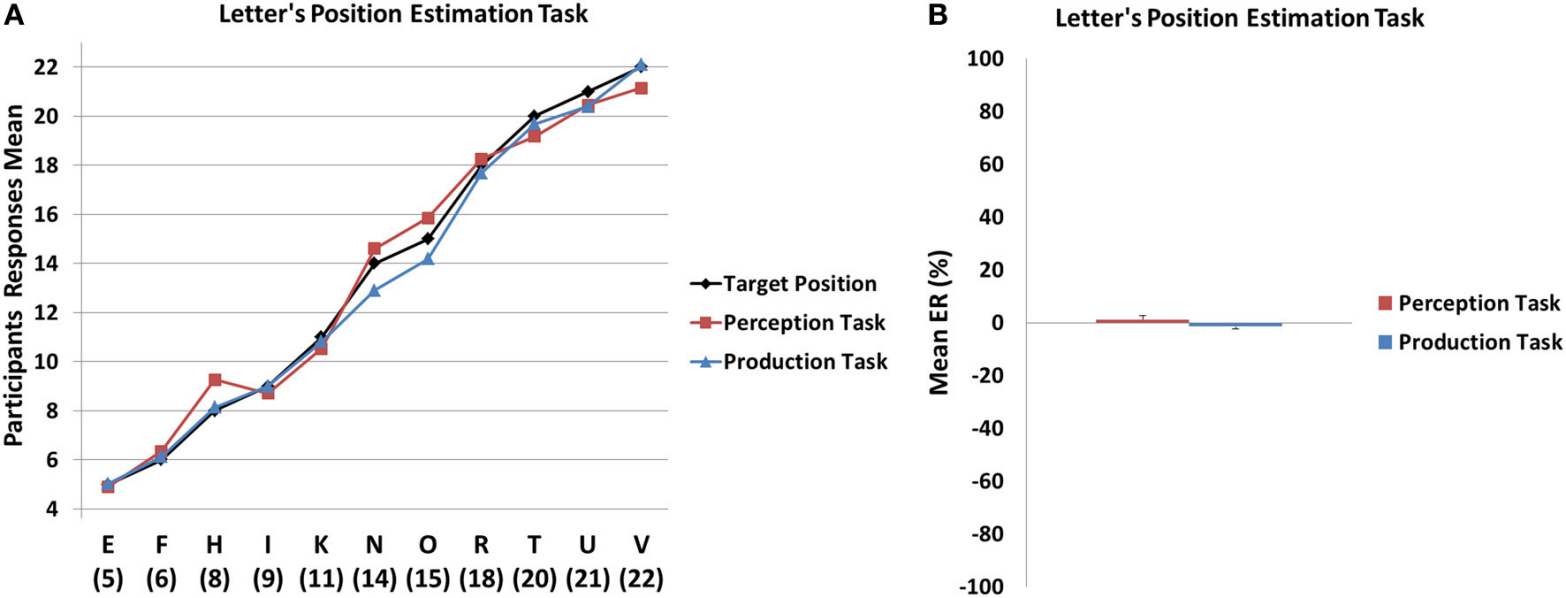
**(A)** Mean estimation values observed in the Letter position perception and production tasks as a function of target values. **(B)** Mean error rate for the Letter position estimation tasks as a function of condition (perception vs. production). No systematic under- or over-estimation were observed in the perception and production tasks. Error bars denote Standard Error of the Mean (SEM).

For the perception task, the results of the LMM showed that the log of the mean estimates increased with the alphabetical position of the letter, *F*_(10, 165)_ = 252.5, *p* < 0.001, while it was not the case for the log of the standard deviation, *F*_(10, 165)_ = 1.2, *ns*. For the production task, both the log of the mean estimates and the log of the standard deviation increased significantly with the alphabetical position, *F*_(10, 165)_ = 470.3, *p* < 0.001 for log(mean); *F*_(10, 165)_ = 4.6, *p* < 0.001 for log(SD); Figure 4.

The mean ER did not significantly differ from zero in the perception [*M* = 1.25 ± 5.2; *t*_(15)_ = 0.95, *p* > 0.3] and production [*M* = −1.49 ± 3.9; *t*_(15)_ = −1.5, *p* > 0.1] conditions, suggesting that the participants did not systematically under- or overestimate the position of the letters. Moreover, the direct comparison of the two tasks revealed that the mean ER from the perception and the production did not significantly differ from each other [*t*_(15)_ = 1.3, *p* > 0.2]. Finally, in order to assess global accuracy, the mean absolute ER was also calculated and, in both perception (*M* = 12.6 ± 5.61) and production (*M* = 9.09 ± 3.98) conditions, these values were significantly different from 0 (all *p* < 0.001).

### Discussion of experiment 2

In contrast to Experiment 1, perceiving and producing an unquantifiable ordered sequence does not lead to systematic under- and over-estimation, respectively. These data therefore suggest that the mere process of perceiving or producing is not sufficient to explain the systematic under- and overestimation patterns observed in Experiment 1.

## General discussion and conclusions

Previous research on numerical cognition has demonstrated that the estimation of numerosity induces either under- or overestimation as a function of task (i.e., perception or production; Castronovo and Seron, 2007; Crollen et al., 2011). Several classical behavioral effects have been reported consistently in numerosity, length, and duration processing (for a recent review, see Dormal and Pesenti, 2012). In order to extend these behavioral data and determine whether numerosity, length, and duration processing involve the same mechanisms, we examined the profile of the error estimation during perception and production judgments of numerosities, lengths, and durations.

In Experiment 1, the pattern of under- and overestimation previously observed during perception and production of numerosity (e.g., Whalen et al., 1999; Cordes et al., 2001; Castronovo and Seron, 2007; Crollen et al., 2011) was extended to the processing of length and duration. Indeed, participants underestimated the length of visually presented horizontal rectangles and the duration of temporal intervals in perception tasks, whereas a systematic overestimation was observed when participants had to produce the length of horizontal rectangles or time intervals. These under- and overestimation patterns were not found in Experiment 2 in which participants had to perceive or produce the alphabetical position of letters, showing that it is not the processes of perceiving or producing something *per se* that cause under- or overestimation. Importantly, the absence of systematic under- and overestimation cannot be accounted by the fact that these perception and production tasks were too easy as demonstrated by the presence of a significant error rates. Together, these findings support the idea that a common metric system, requiring similar mechanisms and/or representations is shared by all the processing of magnitudes (Walsh, 2003; Bueti and Walsh, 2009).

The scalar variability principle predicts imprecise performances, with the average magnitude of the error increasing in proportion to the target (e.g., Meck and Church, 1983). However, this principle cannot explain why these errors correspond to a systematic under- (i.e., negative errors mean) and overestimation (i.e., positive errors mean) in perception and production tasks, respectively. How can this specific pattern of under- and overestimation be accounted for? Underestimation error could arise from a noisy mapping between the objective stimulus magnitude and its mental counterparts (Stevens, 1956; Stevens and Harris, 1962). Consequently, when participants have to produce a quantity, they overestimate their production to compensate their erroneous perception. Overestimation error could also correspond to scalar memory error, which is well documented in the psychophysics of duration memory (Gibbon et al., 1984; Cordes et al., 2001). In the numerical domain, it has been suggested that these opposed patterns of performance could be due to transcoding activities taking place between two differently scaled representations of numerical quantity (Whalen et al., 1999; Castronovo and Seron, 2007; Crollen et al., 2011): a non-symbolic representation assumed to be logarithmically compressed (Dehaene, 2003) on the one hand, and a symbolic numerical representation assumed to be more linear and precise (e.g., Verguts and Fias, 2004; Piazza et al., 2007) on the other hand. According to this bi-directional mapping hypothesis, the participants underestimate numerosities in perception tasks because the symbolic magnitude activated as the output is always smaller than its initial non-symbolic representation. Conversely, in production tasks, participants overestimate numerosities because the non-symbolic output is always larger than the symbolic input. This representational dichotomy has also been highlighted in a recent model, tested through a computational experiment, by postulating the existence of a summation coding (i.e., non-symbolic inputs activate a portion of the mental number line that includes the units smaller than and equal to the target quantities) and a place coding (i.e., symbolic inputs activate their corresponding units and their smaller and larger neighboring units with gradually decreasing strength as a function of distance) system for the processing of numerical magnitude (Verguts and Fias, 2004). These two representation systems are sustained by different cerebral areas (Santens et al., 2010) and do not share the same degree of precision: symbolic magnitudes are represented by sharper (i.e., less variable around the target value) tuning curves than non-symbolic numerosities (Verguts and Fias, 2004; Piazza et al., 2007). In the three perception tasks, the participants had to use symbolic numerical value to realize their estimation of non-symbolic inputs' magnitude; and conversely, participants had to produce non-symbolic outputs corresponding to their estimation of the inputs' magnitude in the production tasks. The systematic distortion found in our results might therefore be due to a general noisy bi-directional mapping between the two types of representations.

Although processing numerosity, length, and duration present some similarities (e.g., global under- and overestimation), the mean overestimation error was significantly smaller in the length production task than in the production of the other two magnitudes, suggesting that participants were more precise in their estimations of length. This observation of better length performance might be accounted for by the fact that humans are confronted to the spatial magnitude earlier and more frequently than to the other magnitudes. Indeed, babies are able to move and use objects in their peripersonal space at a very early stage in their understanding of distance (Piaget and Inhelder, 1948). Although a precise representation of these three magnitudes emerged by 8–9 months of age (e.g., Brannon et al., 2007; Lourenco and Longo, 2010), space may have a basic role as the primary grounding of the general magnitude system (Lourenco and Longo, 2010). Moreover, through geometrical lessons and life experience, it is possible that adults have built a more precise representation of some particular lengths (e.g., 1 m (or 1 yard) corresponding to a step, or 30 cm (1 foot) corresponding to a classic ruler). Finally, despite several methodological precautions (e.g., the use of a large screen of 1.65 x 1.20 m, variation of the presentation position of the rectangle), the participants may have been able to use some spatial cues or strategies to perform better in the length production task. For example, they may have used the lateral edges or the middle of the screen (i.e., corresponding to their position) to guide their length productions, whereas such screen-guided calibrations could probably not occur in the perception task since the lengths were only presented for 250 ms. However, it is worth noting that, even though the length-production performance was better, length was, like the other two magnitudes, generally overestimated.

To conclude, in Experiment 1, under- and overestimation were observed in perception and production tasks, respectively, for numerosity, length, and duration. As the results of Experiment 2 demonstrate that perceiving and producing *per se* were not sufficient to induce this pattern of under- and overestimation, the present findings support the idea of a common metric system underlying the processing of numerosity, length, and duration (Walsh, 2003).

### Conflict of interest statement

The authors declare that the research was conducted in the absence of any commercial or financial relationships that could be construed as a potential conflict of interest.

## Footnotes

^1^Weber–Fechner's law states that Δ *l/l* = *k*, where *l* corresponds to the stimulus intensity and *k* is a constant.

^2^Note that this analysis was also carried out separately for extensive and intensive sets and a significant difference from 0 was found in each condition (extensive: *M* = −37.33 ± 2.74%; *t* (15) = −13.63, *p* < 0.001; and intensive: *M*= −28.30 ± 2.90%; *t* (15)= −9.76, *p* < 0.001).

^3^Similar differences from 0 were observed for the extensive (*M* = 62.73 ± 8.62%; *t*(15) = 7.27, *p* < 0.001) and intensive (*M* = 34.47 ± 7.54%; *t*(15)= 4.57, *p* < 0.001) sets of dots.
